# Residual Vibration Suppression of Piezoelectric Inkjet Printing Based on Particle Swarm Optimization Algorithm

**DOI:** 10.3390/mi15101192

**Published:** 2024-09-26

**Authors:** Huixuan Zhu, Song Li, Runyang Zhu, Feiyang Gao, Zhenyu Yin, Lianqing Liu, Xiongfei Zheng

**Affiliations:** 1 State Key Laboratory of Robotics, Shenyang Institute of Automation, Chinese Academy of Sciences, Shenyang 110016, China; zhuhuixuan@sia.cn (H.Z.);; 2 University of Chinese Academy of Sciences, Beijing 100049, China; 3 Shenyang Institute of Computing Technology, Chinese Academy of Sciences, Shenyang 110168, China; 4 Liaoning Key Laboratory of Domestic Industrial Control Platform Technology on Basic Hardware and Software, Shenyang 110168, China

**Keywords:** piezoelectric inkjet printing, residual vibration suppression, nonlinear hysteresis characteristics, particle swarm optimization algorithm, self-induction principle

## Abstract

Piezoelectric inkjet printing technology, known for its high precision and cost-effectiveness, has found extensive applications in various fields. However, the issue of residual vibration significantly limits its printing quality and efficiency. This paper presents a method for suppressing residual vibration based on the particle swarm optimization (PSO) algorithm. Initially, an improved PI model considering the nonlinear hysteresis characteristics of piezoelectric ceramics is established, and the model is identified through a strain gauge circuit to ensure its accuracy in describing the nonlinear hysteresis characteristics. Subsequently, a dynamic model of the piezoelectric inkjet printing system is constructed, with precise parameter identification achieved using the self-induction principle. This enables precise simulation of residual vibration. Finally, the driving waveform is optimized based on the PSO algorithm, with iterative calculations employed to find the optimal combination of driving waveform parameters, effectively suppressing residual vibration while ensuring sufficient injection energy. The results indicate that this method significantly reduces the amplitude of residual vibration, thereby effectively enhancing printing quality and stability. This research offers a novel solution for residual vibration suppression in piezoelectric inkjet printing technology, potentially advancing its applications in printing and biofabrication.

## 1. Introduction

With the rapid advancement of piezoelectric inkjet printing technology, its applications have expanded into numerous fields, including printing, material forming, and microelectromechanical manufacturing. In recent years, the application of this technology in biofabrication has become particularly prominent [1,2,3,4]. Among these, on-demand inkjet printing (DoD) technology has become a research hotspot due to its ability to finely adjust driving waveforms to control droplet size and velocity [5,6,7,8]. However, the inherent residual vibration problem in the piezoelectric printing process, i.e., the continuous vibration after piezoelectric ceramic actuation severely hinders the improvement of printing accuracy and quality [9,10,11]. Therefore, a deep understanding and effective suppression of the residual vibration phenomenon are crucial for enhancing printing quality.

To deeply understand the residual vibration phenomenon, it is essential to establish a theoretical model of the piezoelectric inkjet system to accurately and comprehensively understand the working mechanism of the printhead to achieve effective vibration control. Regarding model construction, researchers have proposed circuit equivalent and physical models. The circuit-equivalent model transforms the physical process of the piezoelectric inkjet printing system into a circuit model [12]. Researchers have established models based on this method, including a linear time-varying system model based on radial displacement [13], a two-port model [14], and a multi-input, multi-output model [15]. Equivalent circuit models have the advantages of simple structure and ease of analysis. On the other hand, researchers have established physical models based on mechanical principles to describe and analyze the inkjet process. Some studies have established a spring-damping model to describe the oscillation process of piezoelectric ceramics according to the structure of the piezoelectric micro-jetting system [16], combined with the self-sensing technology of the printhead to identify the system model [17,18]. However, these models generally overlook the nonlinear hysteresis characteristics of piezoelectric ceramics, which significantly limits the accuracy of the model simulation. Due to the voltage driving method, the piezoelectric ceramic exhibits hysteresis between the output and input, i.e., the curve corresponding to the increased driving voltage does not coincide with the curve corresponding to the decreased driving voltage [19]. This hysteresis phenomenon also affects the stability of piezoelectric inkjet printing. Therefore, it is necessary to model and analyze the hysteresis characteristics of the piezoelectric printhead to ensure stable printing.

On the other hand, driving waveform optimization is considered a key strategy for suppressing residual vibration and improving printing quality. However, this is a multi-parameter, strongly coupled optimization problem. Traditional methods often rely on manual parameter adjustment. Researchers have optimized driving waveform parameters through manual adjustment using wave propagation theory [20], employing feedforward control methods to suppress residual vibration [21,22,23,24]. However, manual parameter adjustment is inefficient and difficult to achieve the best optimization results. Some researchers have used iterative learning control methods to optimize driving waveform parameters, thereby reducing the impact of residual vibration on printing [25,26]. Iterative learning control methods can automatically adjust parameters and improve optimization efficiency, but they have high computational complexity and are sensitive to parameter changes. In practical applications, it is necessary to comprehensively consider factors such as algorithm efficiency and computational complexity to select a more suitable optimization strategy.

In this study, we established a model that reflects the complex nonlinear hysteresis characteristics of piezoelectric ceramics. Utilizing strain gauge sensors, we achieved the precise identification of this model, thereby ensuring an accurate depiction of these characteristics. Building upon this foundation, we constructed a dynamic model and leveraged the principle of self-induction to precisely determine the model parameters. This enabled us to create a printing system model capable of accurately simulating residual vibrations. Moreover, we introduced a novel residual vibration suppression strategy based on the PSO algorithm. This strategy involved the iterative refinement of the driving waveform, effectively mitigating residual vibrations and substantially enhancing printing performance and efficiency. In summary, this research is anticipated to drive the advancement of piezoelectric inkjet printing technology, offering a more dependable printing solution for critical applications in biofabrication and other fields.

## 2. Materials and Methods

### 2.1. Piezoelectric Inkjet Printing Residual Vibration Detection and Suppression

**Figure 1:**
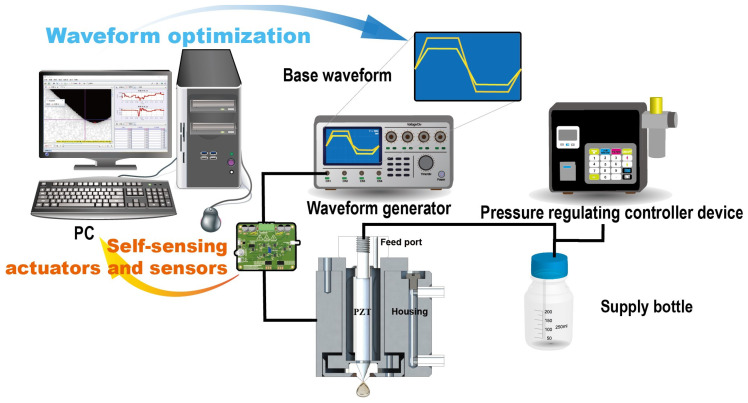
Piezoelectric inkjet printing residual vibration detection and suppression system.

System As shown in Figure 1, the piezoelectric inkjet printing residual vibration detection and suppression system is used. During printing, a basic bipolar waveform is generated by the waveform generator. The bipolar waveform design is used to effectively suppress the system’s residual vibration through the reverse vibration generated by the second wave peak. During the printing process, the pressure controller applies a certain back pressure to the printhead to counteract the surface tension at the nozzle. In this study, the back pressure is maintained in the range of 0.9 to 1.7 kPa. The printhead consists primarily of piezoelectric ceramics, a housing, nozzles, and additional components. The piezoelectric ceramics are securely bonded to the housing using an adhesive. The core component of the printhead is the piezoelectric ceramic (C82, FUJI, Tokyo, Japan). In the inkjet printing process, the piezoelectric ceramic undergoes deformation in response to the driving waveform, thereby producing pressure waves that meticulously control the formation of droplets. After droplet ejection, residual vibration is detected through the piezoelectric ceramic by a self-induction circuit and transmitted to the host computer for processing. The detected data are used to identify the piezoelectric ceramic model, and a simulation system is built based on this model to minimize the system’s residual vibration by optimizing the input waveform.

### 2.2. Nonlinear Hysteresis Compensation Model of Piezoelectric Ceramics

#### 2.2.1. Nonlinear Hysteresis Model of Piezoelectric Ceramics

Due to the non-linear relationship between the input voltage and output displacement of piezoelectric ceramics, the modeling process should consider the nonlinear hysteresis dynamic characteristics of piezoelectric ceramics, which can make the modeling process more accurate and the simulation of printing results more accurate. This paper adopts an improved Prandtl–Ishlinski (PI) nonlinear hysteresis model to express the nonlinear hysteresis effect of piezoelectric ceramics. The classic PI model is characterized as follows [27]: Assuming a piecewise monotonically continuous function set $C_{m}[0,t_{E}]$, the system input $u\left(t\right)\in C_{m}\left[0,t_{E}\right]$, point $0=t_{0}<t_{1}<\cdots <t_{N}=t_{E},$ and the interval $\left[0,t_{E}\right]$ are divided into N sub-intervals $\left[t_{k},t_{k+1}\right]$, where k=0,1,2,⋯,N−1, satisfying that when $t\in [t_{k},t_{k+1}]$, u(t) is a monotonically continuous function with a threshold r≥0. The PI model is:

(1) $$y\left(t\right)=\int_{0}^{+\infty }p\left(r\right)F_{r}\left[u\right]\left(t\right)dr$$

where $p\left(r\right)$ is the integral density function, satisfying $p\left(r\right)\geq 0$ and $\int_{0}^{+\infty }rp\left(r\right)dr<+\infty$. $F_{r}\left[u\right]\left(t\right)$ is the play operator, defined as:

(2) $$\left\{\begin{array}{l} F_{r}\left[u\right]\left(0\right)=f_{r}\left(u\left(0\right),0\right)=w(0)\\ F_{r}\left[u\right]\left(t\right)=f_{r}\left(u\left(t\right),w\left(t_{k}\right)\right);t\in (t_{k},t_{k+1}]\;\mathrm{and}\;0\leq k\leq N-1 \end{array}\right.$$

(3) $$f_{r}\left(u,w\right)=\max\{u-r,\min(u+r,w)\}$$

However, the PI model has many parameters, and the integral part leads to large computational load and truncation error issues. This paper adopts an improved PI model to describe the piezoelectric actuator. In practical applications, the quantization accuracy of the integral density function $p\left(r\right)$ determines the accuracy of the classic PI model, so its computational time complexity and spatial complexity are both $O\left(n\right)$. This section improves the classic PI model algorithmically by transforming the integral operation into a linear mapping, thereby enhancing the computational efficiency of the PI model.

Where $t\in \left[t_{k},t_{k+1}\right]$ and $u\left(t\right)$ is monotonically non-decreasing, the play operator can be rewritten in the form of a piecewise function.

(4) $$F_{r}\left[u\right]\left(t\right)=\left\{\begin{array}{cc} u\left(t\right)-r, & 0\leq r<\frac{u\left(t\right)-u\left(t_{k}\right)}{2}\\ u\left(t_{k}\right)+r, & \frac{u\left(t\right)-u\left(t_{k}\right)}{2}\leq r<\frac{u\left(t_{k+1}\right)-u\left(t_{k}\right)}{2}\\ w\left(t_{k}\right), & r\geq \frac{u\left(t_{k+1}\right)-u\left(t_{k}\right)}{2} \end{array}\right.$$

Then the output is:

(5) $$y\left(t\right)=\int_{0}^{+\infty }p\left(r\right)F_{r}\left[u\right]\left(t\right)dr\;=\int_{0}^{\frac{u\left(t\right)-u\left(t_{k}\right)}{2}}p\left(r\right)(u\left(t\right)-r)dr+\int_{\frac{u\left(t\right)-u\left(t_{k}\right)}{2}}^{\frac{u\left(t_{k+1}\right)-u\left(t_{k}\right)}{2}}p\left(r\right)(u\left(t_{k}\right)+r)dr\;+\int_{\frac{u\left(t_{k+1}\right)-u\left(t_{k}\right)}{2}}^{+\infty }p\left(r\right)w\left(t_{k}\right)dr\;=2\int_{0}^{\frac{u\left(t\right)-u\left(t_{k}\right)}{2}}p\left(r\right)\left(\frac{u\left(t\right)-u\left(t_{k}\right)}{2}-r\right)dr+\int_{0}^{\frac{u\left(t_{k+1}\right)-u\left(t_{k}\right)}{2}}p\left(r\right)(u\left(t_{k}\right)+r)dr\;+\int_{\frac{u\left(t_{k+1}\right)-u\left(t_{k}\right)}{2}}^{+\infty }p\left(r\right)w\left(t_{k}\right)dr$$

Take note of:

(6) $$y\left(t_{k}\right)=\int_{0}^{\frac{u\left(t_{k+1}\right)-u\left(t_{k}\right)}{2}}p\left(r\right)\left(u\left(t_{k}\right)+r\right)dr+\int_{\frac{u\left(t_{k+1}\right)-u\left(t_{k}\right)}{2}}^{+\infty }p\left(r\right)w\left(t_{k}\right)dr$$

So the output is:

(7) $$y\left(t\right)=2\int_{0}^{\frac{u\left(t\right)-u\left(t_{k}\right)}{2}}p\left(r\right)\left(\frac{u\left(t\right)-u\left(t_{k}\right)}{2}-r\right)dr+y\left(t_{k}\right)$$

In the same way, when $t\in \left[t_{k},t_{k+1}\right]$ and $u\left(t\right)$ is monotone non-increasing, output is:

(8) $$y\left(t\right)=-2\int_{0}^{-\frac{u\left(t\right)-u\left(t_{k}\right)}{2}}p\left(r\right)\left(-\frac{u\left(t\right)-u\left(t_{k}\right)}{2}-r\right)dr+y\left(t_{k}\right)$$

Combining the two formulas above, there is:

(9) $$y\left(t\right)=\left\{\begin{array}{c} y\left(0\right),t=0\\ Q\left(u\left(t\right)-u\left(t_{k}\right)\right)+y\left(t_{k}\right),t\in \left(t_{k},t_{k+1}\right]\;\mathrm{and}\;t\in \left(t_{k},t_{k+1}\right],u\left(t\right)\;\mathrm{is}\;\mathrm{non}-\mathrm{decrease}\\ -Q\left(-u\left(t\right)+u\left(t_{k}\right)\right)+y\left(t_{k}\right),t\in \left(t_{k},t_{k+1}\right]\;\mathrm{and}\;t\in \left(t_{k},t_{k+1}\right],u\left(t\right)\;\mathrm{is}\;\mathrm{non}-\mathrm{increasing} \end{array}\right.$$

In the formula:

(10) $$Q\left(x\right)=\int_{0}^{x}p\left(r\right)\left(x-r\right)dr,\;0\leq k\leq N-1.$$

This process transforms the integral term parameters *p*(*r*) of the PI model into linear mapping parameters *Q*(*x*). The parameter function *Q*(*x*) is a mapping function of the input change and output change, thus the new parameter function *Q*(*x*) has a clear physical meaning, is easy to identify, and is convenient to solve, simplifying the complexity of parameter identification and model solution. This model also converts the integral operation of the PI model into a single-value mapping calculation of the input change and output change, reducing the time complexity of PI model calculations from *O*(*n*) to *O*(1), and accurately represents the nonlinear hysteresis characteristics of the piezoelectric ceramic.

#### 2.2.2. Dynamic Model of Piezoelectric Inkjet Printing System

**Figure 2:**
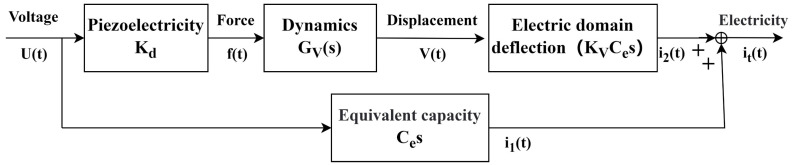
The dynamic model of the printing system.

After constructing the model of the nonlinear hysteresis characteristics of the piezoelectric ceramic, as shown in Figure 2, we developed the dynamic model of the piezoelectric inkjet printing system. Subsequently, these two models were connected in series to simulate the printing results. When the driving voltage is applied to the piezoelectric ceramic, the electric field force acting on the piezoelectric material causes deflection, which alters the thickness of the piezoelectric material. If the system input voltage is $U\left(t\right)$ and the coefficient for the conversion of electric field into force is *K*_d_, then the electric field force exerted on the piezoelectric ceramic is:

(11) $$f\left(t\right)=K_{d}U\left(t\right)$$

Due to the combined effects of the electric field force, elastic stress, and damping force on the piezoelectric domains, the orientation process is inherently an oscillation attenuation process that eventually stabilizes. Macroscopically, this is manifested as the oscillation attenuation process of the piezoelectric ceramic. This process can be expressed using a spring-damped second-order system.

(12) $$m\ddot{x}+c\dot{x}+kx+f\left(t\right)=0$$

(13) $$V\left(t\right)=x$$

In the expression, *f*(*t*) represents the electric field force input, *V*(*t*) represents the deformation output, *m* is the equivalent mass coefficient, *c* is the equivalent damping coefficient, and *k* is the equivalent elastic coefficient. The transfer function of this system is:

(14) $$G_{V}\left(s\right)=\frac{V\left(s\right)}{f\left(s\right)}=\frac{1/m}{s^{2}+(c/m)s+k/m}$$

When the driving waveform *U*(*t*) is applied to the piezoelectric ceramic, the deflection of the piezoelectric actuator causes an additional voltage change $\Delta U\left(t\right)$, which is calculated as $\Delta U\left(t\right){=K}_{V}V\left(t\right)$. In this formula, $K_{V}V$ is the conversion coefficient between the actuator’s deflection and the resulting voltage. Both $U\left(t\right)$ and $\Delta U\left(t\right)$ act on the piezoelectric ceramic’s equivalent capacitance $C_{e}$ to produce the output current $i_{t}$. The relevant equation for this relationship is:

(15) $$Q\left(t\right)=C_{e}\left(U\left(t\right)+\Delta U\left(t\right)\right)$$

(16) $$i_{t}=\frac{dQ\left(t\right)}{dt}=C_{e}\frac{dU\left(t\right)}{dt}+C_{e}K_{V}\frac{d\Delta V\left(t\right)}{dt}$$

In the equation, $C_{e}$ represents the equivalent capacitance of the piezoelectric head, $U\left(t\right)$ is the driving waveform voltage $\Delta U\left(t\right)$ is the voltage change caused by the reorientation of the electric domains, and $Q\left(t\right)$ is the charge. It can be seen that the current is composed of two parts: the first part is $i_{1}=C_{e}\frac{dU\left(t\right)}{dt}$, which is the current generated by the equivalent capacitance. The second part is $i_{2}=C_{e}K_{V}\frac{d\Delta V\left(t\right)}{dt}$, which is the current generated by the piezoelectric effect, with $\Delta U\left(t\right)$ corresponding to the deformation of the piezoelectric ceramic. Therefore, the current equation can be rewritten as:

(17) $$i_{t}=C_{e}\frac{dU\left(t\right)}{dt}+C_{e}K_{V}\frac{dV\left(t\right)}{dt}$$

That is, the current equation can be rewritten as:

(18) $$i_{t}\left(s\right)=\left(C_{e}s\right)U\left(s\right)+\left(K_{V}C_{e}s\right)V\left(s\right)$$

Therefore, the transfer function from the voltage to the current across the piezoelectric ceramic is:

(19) $$G_{I}\left(s\right)=\frac{i_{t}\left(s\right)}{U\left(s\right)}=\frac{\left(K_{V}K_{d}C_{e}/m\right)s}{s^{2}+(c/m)s+k/m}+C_{e}s$$

Having obtained the transfer function of the piezoelectric ceramic, one can utilize this model to simulate the printing results.

### 2.3. Residual Vibration Suppression Optimization Method

**Figure 3:**
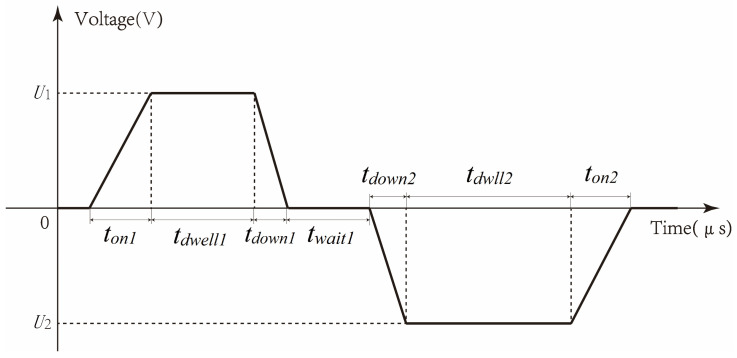
Bipolar voltage excitation waveform.

The use of a bipolar trapezoidal wave as the driving waveform, as shown in Figure 3, operates on the principle of suppressing system vibrations by adding a second wave peak. This paper utilizes the PSO algorithm to achieve the optimization process. The PSO algorithm simulates the foraging behavior of bird flocks, finding the optimal solution to a problem through the cooperation and information sharing of individuals in the group. This method is particularly suitable for solving the residual vibration suppression problem, which is a multi-parameter, strongly coupled optimization problem. This paper optimizes the seven parameters of the driving waveform using the particle swarm algorithm and sets the search range of the corresponding parameters. Since the rated working voltage range of the piezoelectric ceramic is −100 V to 100 V, the optimization process sets the optimization range of U1 and U2 as 0 V to 100 V and −100 V to 0 V, respectively, and the time parameters are set based on factors such as the injection period. The optimization process abstracts each bird as a particle with position and velocity attributes. Each bird adjusts its velocity based on its own and the group’s experience in searching for food. The velocity update formula is as follows:

(20) $$v_{i}^{k+1}=c_{1}v_{i}^{k}+c_{2}\left({pbest}_{i}^{k}-x_{i}^{k}\right)+c_{3}\left({gbest}^{k}-x_{i}^{k}\right)$$

In the formula, k represents the kth iteration, $x_{i}$ denotes the position of the *i*th particle, $v_{i}$ represents the velocity of the ith particle, ${pbest}_{i}$ signifies the historical best value of the ith particle, and gbest represents the historical best value of all particles. The coefficients $c_{1}$, $c_{2}$, $c_{3}$ correspond to the inertia weight coefficient, the self-learning factor coefficient, and the global learning factor coefficient, respectively.

The optimization goal of residual vibration suppression can be characterized by a cost function. The objective is to increase the height of the first effective output peak to achieve injection energy and reduce the other peaks to suppress residual vibration. The cost function is defined based on this goal as follows:

(21) $$cost=Ay_{peak}\left(1\right)+By_{peak}\left(2\right)+\sum_{i=3}^{\infty }y_{peak}\left(i\right)$$

In the equation, $y_{peak}\left(i\right)$ represents the absolute value of the ith peak of the output waveform, and A and B are constants. By optimizing this cost function, the best combination of driving waveform parameters can be found, which minimizes residual vibration while ensuring the height of the first effective output peak.

## 3. Results and Discussion

### 3.1. Identification of PI Nonlinear Hysteresis Model 

**Figure 4:**
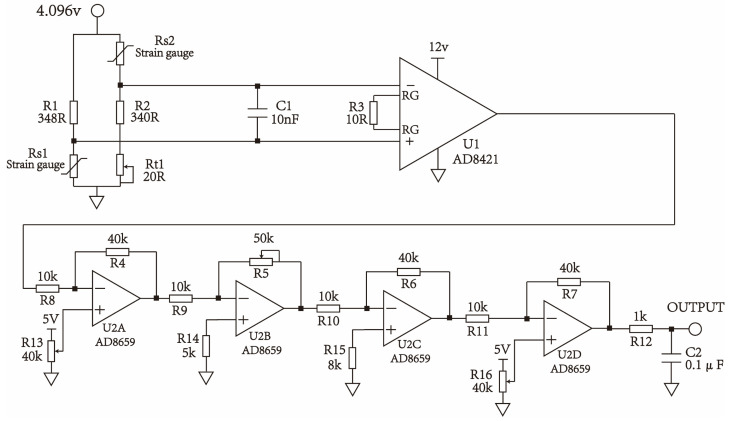
Strain gauge detection circuit.

After establishing the system model, the PI nonlinear hysteresis model is identified first. The strain gauge is bonded to the piezoelectric ceramic, which is not integrated into the print head, to measure its deformation in an offline manner. The results obtained from this offline identification method are essentially consistent with those measured in an integrated nozzle setup, facilitating a more convenient experimental process without affecting the vibration of the piezoelectric ceramic during printing. The detection circuit is shown in Figure 4. The strain gauges Rs1 and Rs2, resistors R1 and R2, and the potentiometer RT1 form a two-arm Wheatstone bridge, which is used to detect small changes in resistance. The strain gauge Rs1 is attached to the piezoelectric ceramic to measure its deformation, while Rs2 is configured with the same attributes and wiring as Rs1 to compensate for temperature and line interference. The resistance value of the strain gauges is 350 Ω, and the potentiometer Rt1 is used to calibrate the static error of the strain gauges and resistors. U1 is an instrumentation amplifier with a high-impedance characteristic that is very suitable for the primary amplification of the bridge signal. Operational amplifiers U2A to U2D constitute a four-stage amplification circuit. U2A is used for signal zeroing for subsequent processing; U2B is an amplitude modulation circuit that controls the overall gain of the acquisition system; U2D is a zeroing circuit used to shift the signal within the input range of the ADC chip.

The strain gauge measures the deformation of the piezoelectric ceramic, and its detection circuit is shown in Figure 4. A double-arm Wheatstone bridge is used as the resistance transmission link, and an instrumentation amplifier is used as the preamplifier, with multi-stage amplifiers for zero adjustment.

**Figure 5:**
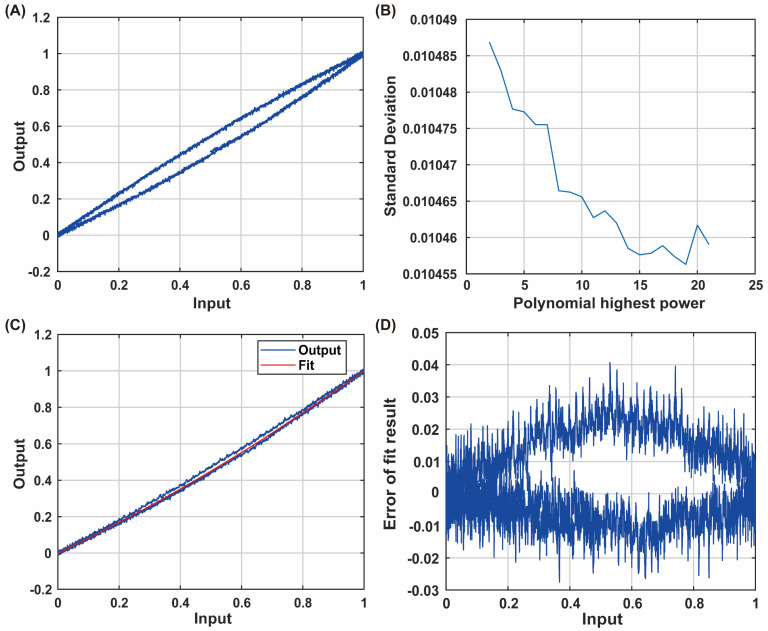
Identification of the PI nonlinear hysteresis system. (**A**) Input–output hysteresis waveform. (**B**) Relationship between polynomial degree and standard deviation. (**C**) Comparison of fitting results with hysteresis loop shape. (**D**) Fitting error.

To minimize the influence of frequency during the collection of hysteresis characteristics, we tested at a lower frequency, with an input voltage of y = 90sin(t). The input and output time-domain waveforms and hysteresis loop are shown in Figure 5A. From the figure, it can be observed that one input of the piezoelectric ceramic can correspond to multiple outputs, demonstrating its hysteresis characteristics.

A polynomial is used to fit the hysteresis:

(22) $$y=a_{0}+a_{1}x+a_{2}x^{2}+\ldots +a_{n}x^{n}$$

To determine the highest degree n, the square error of different highest degrees is calculated, as shown in Figure 5B. When n is greater than 8, the trend of the square error reduction slows down, so n = 8 is selected to avoid overfitting and underfitting. The identification results of the model are A = [a8, a7, …, a0] = [19.014, −75.155, 120.8, −101.21, 46.92, −11.697, 1.5782, 0.74743, 0]. As shown in Figure 5C, the red polynomial fit and the blue hysteresis loop show that the fit curve and the measured curve are coincident, as shown in Figure 5D, with an error of about 2%, indicating that the fitted polynomial can accurately reflect the nonlinear hysteresis characteristics of the piezoelectric ceramic.

### 3.2. Identification of Dynamic Model of Piezoelectric Printing System

**Figure 6:**
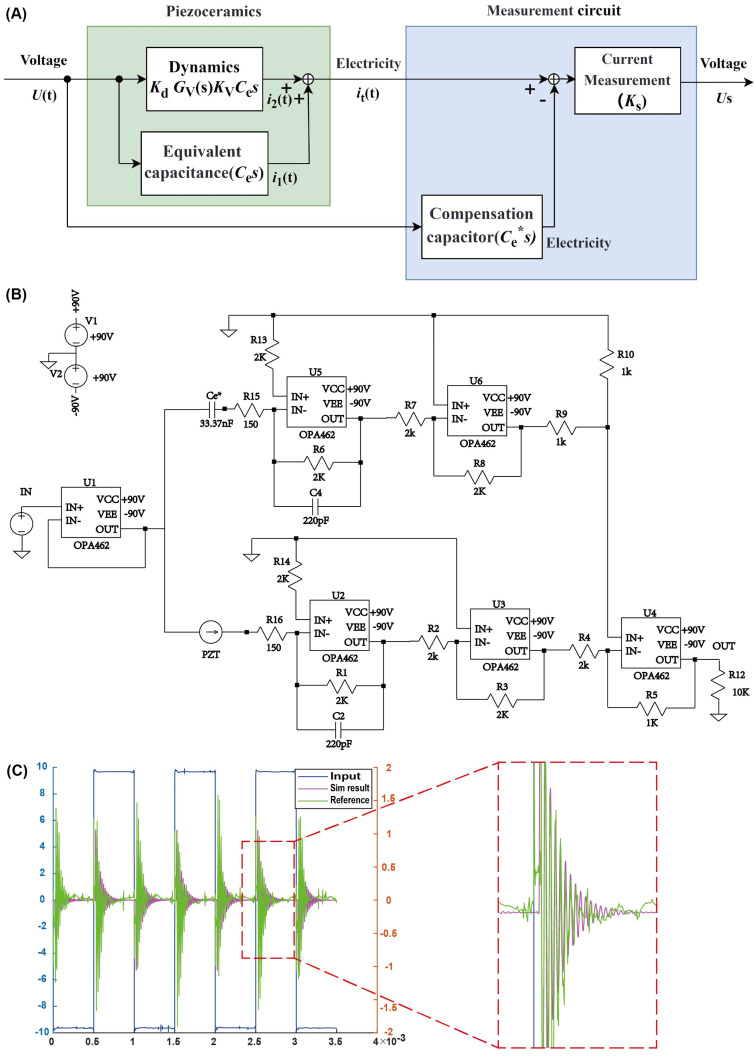
Identification of dynamic model. (**A**) Structure diagram of the self-induction residual vibration acquisition system. (**B**) Circuit diagram of the self-induction residual vibration acquisition system. (**C**) Model identification results.

To identify the dynamic model of the piezoelectric printing system, the self-induction principle is used, and the identification system is shown in Figure 6A. The system model uses data from the self-inductive circuit for model identification, and the piezoelectric effect detects the final result of pressure wave propagation in the fluid, which is the outcome after fluid-structure interaction. Although the fluid–structure interaction process is simplified in the modeling, the identification process through the piezoelectric effect detects the effect of fluid-structure interaction, so the model also reflects certain properties of the ink and fluid-structure interaction to some extent. Based on the principle of self-induction, the self-inductive circuit shown in Figure 6B is capable of identifying the current in the piezoelectric ceramic. This current is produced by the interaction between pressure waves and the piezoelectric ceramic, which in turn reflects the ejection and perturbation of droplets. The measurement circuit includes multiple high-voltage power amplifiers, forming a current subtractor and a current-to-voltage converter. By compensating for the equivalent capacitance of the piezoelectric ceramic, the output of the measurement circuit only contains structural deformation information, which records the vibration process of the piezoelectric ceramic, including the main waveform information and residual vibration information. The identification results of the system are shown in Figure 6C, with a small error between the simulation and the actual measurement, verifying the accuracy of the model.

The transfer function of the system after adding the identification system is:

(23) $$G_{s}\left(s\right)=\frac{U_{s}\left(s\right)}{U\left(s\right)}=\frac{\left(K_{s}K_{V}K_{d}C_{e}/m\right)s}{s^{2}+(c/m)s+k/m}=\frac{b_{1}s}{s^{2}+a_{1}s+a_{0}}$$

The identification results are *a*_0_ = 1.055 × 10^11^, *a*_1_ = 3.046 × 10^4^, *b*_1_ = 1.907 × 10^4^. To enhance the versatility of the model, it does not specify the exact values of the piezoelectric ceramic’s parameters but instead employs identification techniques to determine these values. Consequently, specific parameters for the C82 ceramic are not incorporated into the model. Instead, the relevant parameters are identified through the identification process. When employing different piezoelectric ceramics, the structure of the model remains unchanged and is equally applicable to other types. Due to the varying characteristics of different piezoelectric ceramics, only the model needs to be re-identified with the new material, and the identification method remains applicable. Only the parameters obtained through the identification process will vary.

### 3.3. Residual Vibration Suppression Optimization Based on Particle Swarm Algorithm 

**Figure 7:**
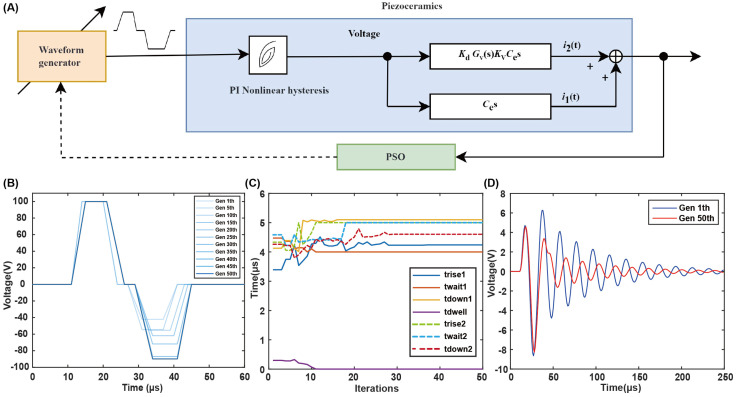
Residual vibration suppression optimization based on particle swarm algorithm. (**A**) Structure diagram of the particle swarm waveform optimization system. (**B**) Iterative optimization of the driving waveform. (**C**) The iterative process of residual vibration suppression optimization for driving waveform time parameters. (**D**) Comparison of residual vibration suppression effects of the optimized output waveform.

This study uses PSO arithmetic for the suppression of residual vibrations, involving the computational optimization of seven parameters associated with the bipolar waveform. The goal is to find the best combination of driving waveform parameters. As shown in Figure 7A, the particle swarm waveform optimization system structure diagram is shown. Initially, the waveform generator generates a bipolar waveform, which, through the piezoelectric ceramic PI nonlinear hysteresis model and the dynamic model, forms the printing system’s residual vibration output. After PSO analysis, the parameters of the waveform generator are controlled to achieve residual vibration suppression. The waveform is shown in Figure 7B, illustrating the iterative optimization process of the driving waveform over 50 generations of the particle swarm algorithm. As shown in Figure 7C, the optimization process of the time parameters of the driving waveform is shown, with the parameters gradually approaching the optimal values as the iteration progresses. As shown in Figure 7D, the blue curve represents the energy and residual vibration effect of the waveform in the 1st generation, while the red curve represents the energy and residual vibration effect of the waveform in the 50th generation. From the figure, it can be seen that the energy of the first peak of the two waveforms is almost the same, both providing sufficient energy for droplet ejection. However, the blue waveform has a very large residual vibration, with the second residual vibration peak even exceeding the first main energy peak, which easily leads to printing problems such as satellite droplets, seriously affecting printing quality. In contrast, the red waveform has significantly smaller residual vibration, with the second peak energy far less than the first main peak, and the total energy of subsequent residual vibrations has been effectively suppressed. By comparison, the residual vibration suppression method based on particle swarm driving proposed in this paper can effectively suppress residual vibration and provide a strong guarantee for efficient and stable inkjet printing. The PSO algorithm can be applied to the resonance suppression problem in other piezoelectric inkjet printing systems. It is applicable to different types of piezoelectric inkjet printing systems, including diaphragm-type and tube-type piezoelectric printing. The model establishment and algorithm optimization do not impose specific constraints on the shape and structure of the piezoelectric ceramics, making it suitable for various systems. The PSO algorithm is also applicable to other types of multi-objective, strongly coupled complex optimization problems.

## 4. Conclusions

This study addresses the common problem of residual vibration in piezoelectric inkjet printing systems, proposing an innovative solution based on the PSO algorithm. By constructing and identifying an improved PI model and dynamic model that accurately reflects the system’s nonlinear hysteresis characteristics, a mathematical model is established, laying the theoretical foundation for effective residual vibration suppression. Furthermore, the residual vibration suppression strategy based on the PSO algorithm, which optimizes driving waveform parameters, effectively reduces the amplitude of residual vibration, significantly improving printing quality and stability, thus providing a guarantee for the practical application of piezoelectric inkjet printing technology. If alternative inks or printing systems are employed, it is merely necessary to reapply the method described herein for model identification. Once new model parameters are obtained, the particle swarm optimization algorithm can still be utilized for the research on residual vibration suppression. In the future, we will focus on developing more efficient optimization algorithms and exploring the residual vibration characteristics of different printing materials and modes to build more accurate simulation models and more universal suppression strategies. In addition, we will actively promote the application of this method in other inkjet printing technologies such as thermal inkjet printing, contributing to the overall advancement and industrial upgrading of inkjet printing technology.

## Data Availability

Data are available from the corresponding author upon reasonable request.

## References

[1] Roh J., Kim H., Park M., Kwak J., Lee C. (2017). Improved Electron Injection in All-Solution-Processed n-Type Organic Field-Effect Transistors with an Inkjet-Printed ZnO Electron Injection Layer. Appl. Surf. Sci..

[2] Li X., Liu B., Pei B., Chen J., Zhou D., Peng J., Zhang X., Jia W., Xu T. (2020). Inkjet Bioprinting of Biomaterials. Chem. Rev..

[3] Kwon J., Takeda Y., Shiwaku R., Tokito S., Cho K., Jung S. (2019). Three-Dimensional Monolithic Integration in Flexible Printed Organic Transistors. Nat. Commun..

[4] Li X., Chen J., Liu B., Wang X., Ren D., Xu T., Ovsianikov A., Yoo J., Mironov V. (2018). Inkjet Printing for Biofabrication. 3D Printing and Biofabrication.

[5] Aqeel A.B., Mohasan M., Lv P., Yang Y., Duan H. (2020). Effects of the Actuation Waveform on the Drop Size Reduction in Drop-on-Demand Inkjet Printing. Acta Mech. Sin..

[6] Zhu H., Li R., Li S., Guo K., Ji C., Gao F., Zheng Y., Zhu R., Wang H., Zhang L. (2024). Multi-Physical Field Control Piezoelectric Inkjet Bioprinting for 3D Tissue-like Structure Manufacturing. Int. J. Bioprint..

[7] Peng J., Huang J., Wang J., Meng F., Gong H., Ping B. (2022). The Driving Waveform Design Method of Power-Law Fluid Piezoelectric Printing Based on Iterative Learning Control. Sensors.

[8] Li E.Q., Xu Q., Sun J., Fuh J.Y.H., Wong Y.S., Thoroddsen S.T. (2010). Design and Fabrication of a PET/PTFE-Based Piezoelectric Squeeze Mode Drop-on-Demand Inkjet Printhead with Interchangeable Nozzle. Sens. Actuators A Phys..

[9] Li H., Liu J., Li K., Liu Y. (2019). Piezoelectric Micro-Jet Devices: A Review. Sens. Actuators A Phys..

[10] Zhang Y., Hu G., Liu Y., Wang J., Yang G., Li D. (2022). Suppression and Utilization of Satellite Droplets for Inkjet Printing: A Review. Processes.

[11] Li H., Yang W., Duan Y., Chen W., Zhang G., Huang Y., Yin Z. (2022). Residual Oscillation Suppression via Waveform Optimization for Stable Electrohydrodynamic Drop-on-Demand Printing. Addit. Manuf..

[12] Gallas Q., Sheplak M., Kaysap A., Carroll B., Nishida T., Cattafesta L., Mathew J., Holman R. Lumped Element Modeling of Piezoelectric-Driven Synthetic Jet Actuators. Proceedings of the 40th AIAA Aerospace Sciences Meeting & Exhibit.

[13] Wang J., Huang J., Peng J. (2019). Hydrodynamic Response Model of a Piezoelectric Inkjet Print-Head. Sens. Actuators A Phys..

[14] Shah M.A., Lee D.G., Hur S. (2019). Design and Characteristic Analysis of a MEMS Piezo-Driven Recirculating Inkjet Printhead Using Lumped Element Modeling. Micromachines.

[15] Khalate A.A., Bombois X., Ye S., Babuška R., Koekebakker S. (2012). Minimization of Cross-Talk in a Piezo Inkjet Printhead Based on System Identification and Feedforward Control. J. Micromech. Microeng..

[16] Oktavianty O., Kyotani T., Haruyama S., Kaminishi K. (2019). New Actuation Waveform Design of DoD Inkjet Printer for Single and Multi-Drop Ejection Method. Addit. Manuf..

[17] Kwon K.-S., Kim W. (2007). A Waveform Design Method for High-Speed Inkjet Printing Based on Self-Sensing Measurement. Sens. Actuators A Phys..

[18] Wang J., Huang J., Peng J., Zhang J. (2019). Piezoelectric Print-Head Drive-Waveform Optimization Method Based on Self-Sensing. Sens. Actuators A Phys..

[19] Rakotondrabe M., Clévy C., Lutz P. (2010). Complete Open Loop Control of Hysteretic, Creeped, and Oscillating Piezoelectric Cantilevers. IEEE Trans. Automat. Sci. Eng..

[20] Bogy D.B., Talke F.E. (1984). Experimental and Theoretical Study of Wave Propagation Phenomena in Drop-on-Demand Ink Jet Devices. IBM J. Res. Dev..

[21] Khalate A.A., Bombois X., Babuška R., Wijshoff H., Waarsing R. (2011). Performance Improvement of a Drop-on-Demand Inkjet Printhead Using an Optimization-Based Feedforward Control Method. Control Eng. Pract..

[22] Khalate A.A., Bombois X., Scorletti G., Babuska R., Koekebakker S., de Zeeuw W. (2012). A Waveform Design Method for a Piezo Inkjet Printhead Based on Robust Feedforward Control. J. Microelectromech. Syst..

[23] Shah M.A., Lee D.-G., Lee B.Y., Kim N.W., An H., Hur S. (2020). Actuating Voltage Waveform Optimization of Piezoelectric Inkjet Printhead for Suppression of Residual Vibrations. Micromachines.

[24] Nguyen V.-T., Leong J.Y.C., Watanabe S., Morooka T., Shimizu T. (2021). A Multi-Fidelity Model for Simulations and Sensitivity Analysis of Piezoelectric Inkjet Printheads. Micromachines.

[25] Wassink M.G., Bosch N.J.M., Bosgra O.H., Koekebakker S. Enabling Higher Jet Frequencies for an Inkjet Printhead Using Iterative Learning Control. Proceedings of the 2005 IEEE Conference on Control Applications 2005—CCA 2005.

[26] Wang J., Xiong C., Huang J., Peng J., Zhang J., Zhao P. (2023). Waveform Design Method for Piezoelectric Print-Head Based on Iterative Learning and Equivalent Circuit Model. Micromachines.

[27] Krasnosel’skiǐ M.A., Pokrovskiǐ A.V. (1989). Systems with Hysteresis.

